# Calebin A, a Compound of Turmeric, Down-Regulates Inflammation in Tenocytes by NF-κB/Scleraxis Signaling

**DOI:** 10.3390/ijms23031695

**Published:** 2022-02-01

**Authors:** Anna-Lena Mueller, Aranka Brockmueller, Ajaikumar B. Kunnumakkara, Mehdi Shakibaei

**Affiliations:** 1 Musculoskeletal Research Group and Tumor Biology, Chair of Vegetative Anatomy, Institute of Anatomy, Faculty of Medicine, Ludwig-Maximilian-University Munich, Pettenkoferstr. 11, D-80336 Munich, Germany; A.Mueller@med.uni-muenchen.de (A.-L.M.); aranka.brockmueller@med.uni-muenchen.de (A.B.); 2 Cancer Biology Laboratory and DBT-AIST International Center for Translational and Environmental Research (DAICENTER), Department of Biosciences and Bioengineering, Indian Institute of Technology (IIT) Guwahati, Guwahati 781039, India; kunnumakkara@iitg.ac.in

**Keywords:** tenocytes, Calebin A, tendinitis microenvironment, T-lymphocytes, cytokines, NF-κB, scleraxis

## Abstract

Calebin A (CA) is one of the active constituents of turmeric and has anti-inflammatory and antioxidant effects. Excessive inflammation and cell apoptosis are the main causes of tendinitis and tendinopathies. However, the role of CA in tendinitis is still unclear and needs to be studied in detail. Tenocytes in monolayer or 3D-alginate cultures in the multicellular tendinitis microenvironment (fibroblast cells) with T-lymphocytes (TN-ME) or with TNF-α or TNF-β, were kept without treatment or treated with CA to study their range of actions in inflammation. We determined that CA blocked TNF-β-, similar to TNF-α-induced adhesiveness of T-lymphocytes to tenocytes. Moreover, immunofluorescence and immunoblotting showed that CA, similar to BMS-345541 (specific IKK-inhibitor), suppressed T-lymphocytes, or the TNF-α- or TNF-β-induced down-regulation of Collagen I, Tenomodulin, tenocyte-specific transcription factor (Scleraxis) and the up-regulation of NF-κB phosphorylation; thus, its translocation to the nucleus as well as various NF-κB-regulated proteins was implicated in inflammatory and degradative processes. Furthermore, CA significantly suppressed T-lymphocyte-induced signaling, similar to TNF-β-induced signaling, and NF-κB activation by inhibiting the phosphorylation and degradation of IκBα (an NF-κB inhibitor) and IκB-kinase activity. Finally, inflammatory TN-ME induced the functional linkage between NF-κB and Scleraxis, proposing that a synergistic interaction between the two transcription factors is required for the initiation of tendinitis, whereas CA strongly attenuated this linkage and subsequent inflammation. For the first time, we suggest that CA modulates TN-ME-promoted inflammation in tenocytes, at least in part, via NF-κB/Scleraxis signaling. Thus, CA seems to be a potential bioactive compound for the prevention and treatment of tendinitis.

## 1. Introduction

Tendinopathies is a medical term describing various types of tendon injuries, including trauma, rupture, and especially inflammation-related tendinitis, which are occurring more frequently worldwide [1,2] and account for almost one-third of all musculoskeletal consultations [3]. Moreover, tendinopathies are disabling but also very painful conditions that significantly affect the quality of patients’ daily lives [4,5], explaining their strong desire for relief. The standard medical treatment commonly provided to patients comprises oral non-steroidal anti-inflammatory drugs (NSAIDs) [6] and corticosteroid injections [7]. However, their efficacy in reducing pain and inflammation comes with high costs, as these treatments are associated with serious side effects in multiple organs such as the gastrointestinal, cardiovascular, hepatic, renal, cerebral and pulmonary systems [8,9,10]. In addition, a link was shown between NSAIDs application and inhibitory effects on proteoglycan synthesis and tendon cell proliferation [11], as well as the attenuation of collagen during corticosteroid treatment [12,13,14]. These facts emphasize the urgency for a safer and more specific therapy in the treatment of tendinopathies.

The reasons underlying tendinopathies are various and partly go hand in hand, such as the natural process of aging, physical inactivity and a sedentary lifestyle, mechanical stress and overuse, genetic predisposition or a poor diet [15,16], to name just a few. On the other hand, research showed that tendon ruptures and tendinitis can also occur as a consequence of high-performance sports [17,18] or as side effects of certain drug therapies, such as the antibiotic group of quinolones [19,20,21], primarily by destroying the connection between tenocytes and the extracellular matrix (ECM).

Morphologically, tenocytes have a fibroblast-like shape, expressing Scleraxis as a tendon-specific transcription factor [22,23], and they are embedded in the ECM, a complex and highly dynamic network composed of non-cellular components such as collagen (mainly Collagen I), Elastin, Fibronectin and proteoglycans. The ECM, produced by tenocytes themselves, not only determines a tendon’s stability and its mechanical properties, but is also essential for the regulation of cellular functions such as growth, survival, differentiation or the maintenance of homeostasis. These regulatory processes are mediated by cell surface receptors such as the integrin family, which transmit signals from ECM to cells and vice versa [24,25,26,27,28].

In this context, several studies showed that integrins, as heterodimeric transmembrane protein receptors, play a fundamental role in this process. They not only integrate ECM and cytoskeletal structures into a functional unit, but also actively transduce signals into the cell and vice versa, consequently possessing a wide range of functions essential for tissue survival, differentiation, and homeostasis [29,30,31,32]. Recalling the importance of the various functions of integrin and its role of being strongly involved in ECM anchorage signals, thus the survival of cells, it becomes clear that, the disruption of the specific interplay between ECM and cells might cause apoptotic cell death [32,33].

To date, inflammation and pro-inflammatory processes are known to primarily contribute to the pathogenesis of tendinopathy and tendinitis [25], thus being accompanied by catabolic events, ultrastructural changes, and even tenocyte apoptosis. The up-regulation of pro-inflammatory cytokines such as TNF-α or TNF-β, and the activation of catabolic enzymes such as Cyclooxygenase-2 (COX-2) or Matrix-metalloproteinase (MMP) accompanying inflammatory processes, are mostly promoted by the activation of the Nuclear Factor κappa B (NF-κB) pathway, a pro-inflammatory transcription factor engaged in the regulation of various genes [32,34,35,36]. NF-κB usually resides in the cytoplasm of cells, but when activated through its phosphorylation, it translocates into the nucleus. Since NF-κB serves as a central mediator for inflammatory processes and its up-regulation is mostly associated with inflammatory disease, it represents a potential target in treatment strategies [37,38,39].

Recently, the attention towards natural products as promising and effective treatment options has grown, especially in inflammation-triggered disease, since many studies clearly demonstrated their multi-targeting potential and the anti-inflammatory evidence of polyphenols, which is partially and specifically achieved by inhibiting NF-κB [40,41,42,43,44,45]. In fact, in contrast to standard therapy, natural compounds provide a much safer and less expensive treatment with no undesirable side effects [46].

Calebin A (CA) [4-(3-methoxy-4-hydroxyphenyl)-2-oxo-3-enebutanyl 3-(3-methoxy-4-hydroxyphenyl) propenoate], a non-curcuminoid active component obtained from the rhizome of turmeric (Curcuma longa L., Zingiberaceae) [47,48] is described to possess anti-inflammatory, anti-tumor and anti-oxidant properties [47,49,50,51], whereby many of these studies identified a significant suppressing effect of CA on the activation of the pro-inflammatory NF-κB pathway. However, most of the studies were based on the investigation of tumor cells, and only a few were carried out on cells of the connective tissue [49,51,52,53].

To our knowledge, there are currently no studies that investigate the impact of CA on tenocytes and its potential inhibitory effect on NF-κB and on the tendon-specific transcription factor Scleraxis in an inflammatory environment. Therefore, the present study aimed to investigate the mechanism of action of CA and its potential to alleviate inflammation by targeting the NF-κB/Scleraxis signaling pathway in a tendinitis-like environment in vitro.

## 2. Results

**Figure 1:**
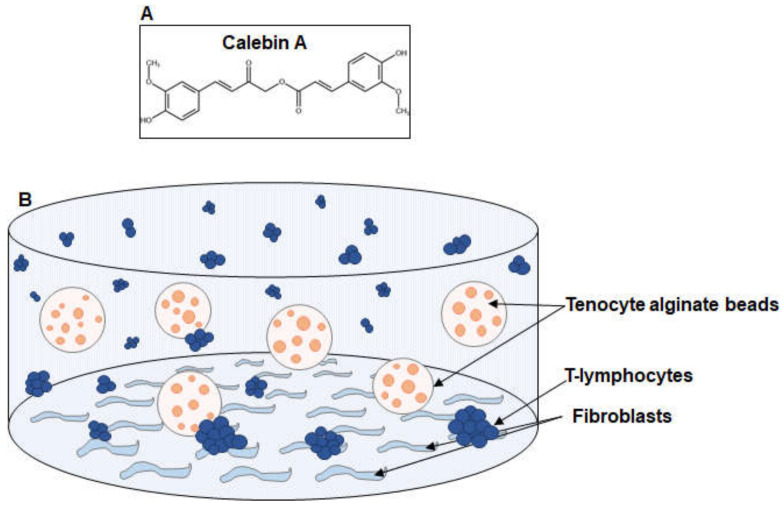
(**A**) Chemical structure of the natural compound Calebin A. (**B**) Graphic illustration of the experimental set-up for creation of pro-inflammatory multicellular microenvironment tendinitis (TN-ME) conditions in alginate cultures.

The aim of this study was to investigate the potential anti-inflammatory effects of Calebin A (Figure 1A) on tenocytes in an inflammatory environment and to examine the specific mechanism by which CA, as an active ingredient, exerts its effects on tendon tissue. To this end, an inflammatory environment was created in tenocytes monolayer cultures as well as in an in vivo tendinitis-like 3D-alginate culture (TN-ME) model (Figure 1B).

### 2.1. Calebin A Suppresses TNF-β- Similar to TNF-α-Mediated Adhesiveness of T-Lymphocytes to Tenocytes

It is well known that inflammatory processes in tissues are associated with the recruitment and migration of T-lymphocytes and other leukocytes to the site of inflammation [54]. To investigate whether a similar effect can be detected in tenocytes, we performed an adhesion experiment with T-lymphocytes. Tenocytes were grown to subconfluence in monolayer cultures, either by themselves or in co-cultures with T-lymphocytes, treated as outlined in Material and Methods.

**Figure 2:**
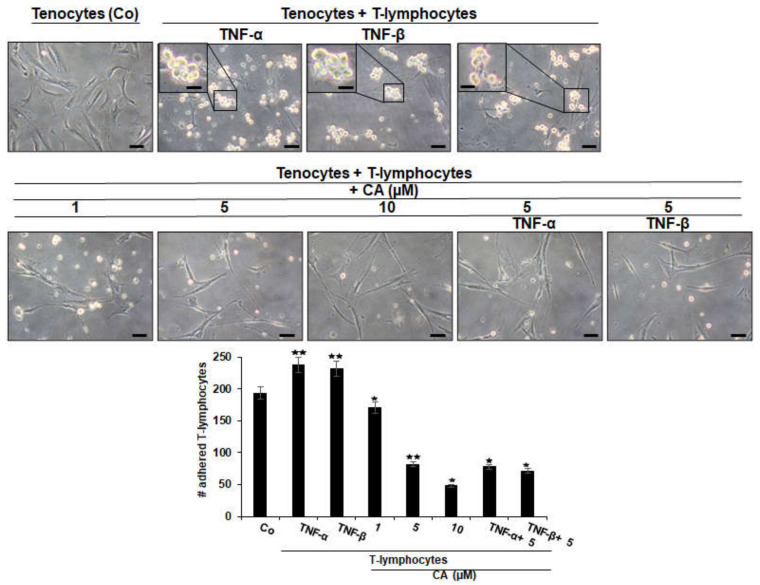
Effect of Calebin A (CA), TNF-α or TNF-β on T-lymphocytes adhesiveness on tenocytes in an inflammatory environment. Serum-starved tenocyte monolayer cultures alone (control, Co), or co-cultured with T-lymphocytes, or co-cultured with T-lymphocytes and TNF-α or TNF-β (10 ng/mL), were either left untreated or treated with various concentrations of CA (1, 5, 10 µM) for 18 h and, subsequently, photographed using a light microscope (Zeiss, Jena, Germany). All assays were performed three times. Data with *p* < 0.05 (*) and *p* < 0.01 (**) indicate a significant difference compared to control. Magnification: ×100; scale bar = 30 nm. Insets: magnification: ×400; scale bar = 3 nm.

Co-culturing tenocytes with T-lymphocytes alone (as control, Co) or with additional treatment with cytokines (such as TNF-α or TNF-β) showed the similar tendency of T-lymphocytes to attach and adhere to tenocytes, which is significantly increased by cytokine stimulation, underlining their role in enhancing T-lymphocyte migration, thus manifesting an inflammatory environment (Figure 2). In contrast, untreated co-cultures, or co-cultures treated with CA or with CA and pro-inflammatory cytokines, showed a remarkable suppression of T-lymphocyte accumulation and adherence to tenocytes with a concentration-dependent effect (Figure 2). Overall, these results indicate the adhesion of T-lymphocytes to tenocytes in a tendinitis environment as one of the main targets of CA for its anti-inflammatory mechanisms in inflammatory environments in tenocyte culture.

### 2.2. Calebin A Suppresses T-Lymphocyte-Induced NF-κB Phosphorylation/Nuclear Translocation and Down-Regulation of Scleraxis

**Figure 3:**
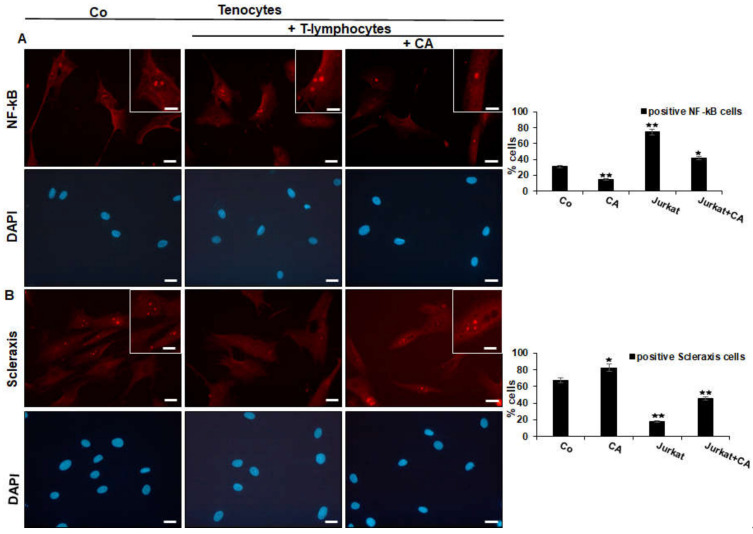
Effect of Calebin A (CA) on NF-κB (**A**) and on Scleraxis (**B**) in tenocytes cultured in an inflammatory environment analyzed by immunofluorescence. Serum-starved tenocyte monolayer cultures alone (control, Co) or co-cultured with T-lymphocytes were either left untreated or treated with CA (5 µM) for 5 h. Magnification ×400; scale bar = 45 nm. All experiments were conducted three times and quantification of positively NF-κB- or Scleraxis-labeled nuclei was performed by counting 400–500 cells from 10 different microscopic fields. Values of *p* < 0.05 (*), *p* < 0.01 (**) were considered statistically significant in relation to control.

To analyze whether the above observations of enhanced or suppressed adhesiveness might be associated with the up-regulation of either inflammatory-specific or the down-regulation of tendon-specific proliferating signaling in tenocytes, we first performed an immunofluorescence analysis with the master regulator of inflammation NF-κB and the tendon-specific transcription factor, Scleraxis. Tenocytes grown in monolayer on glass plates were either cultured alone (Co) or grown in T-lymphocyte co-cultures and left untreated or treated with CA, as precisely described in Material and Methods. The immunolabeling of the tenocyte control culture showed a moderate expression of NF-κB, while co-culturing with T-lymphocytes resulted in a two-fold increase in NF-κB phosphorylation, and thus the translocation into the nucleus of tenocytes, where it may modulate gene expression. Interestingly, the addition of a moderate concentration of CA to the tenocyte co-culture, clearly showed a down-regulation of NF-κB phosphorylation and a translocation to the nucleus of tenocytes comparable to the initial condition (Co) (Figure 3A). Altogether, these data indicate that CA is able to down-regulate T-lymphocyte-enhanced expression and nuclear translocation of p65-NF-κB to tenocytes in the tendinitis environment, which is at least one of the major mechanisms for its anti-inflammatory effect in tendinitis.

To examine whether the inflammation-protective effect of CA in tenocytes is not only linked with blocking NF-κB activation, but also with triggering tendogenic transcription factor Scleraxis, immunofluorescence labeling against Scleraxis was performed in the same experimental set-up as used for NF-κB (Figure 3B). In the reference control, tenocytes revealed intensive fluorescent labeling of Scleraxis in the nucleus, which was markedly reduced to almost no signaling in the co-culture with T-lymphocytes. In the presence of CA, a strong signaling of Scleraxis was exhibited again in tenocytes co-cultured with T-lymphocytes. Overall, these results suggest that CA may act as an actively multi-targeting component, exerting its effects, at least in part, by suppressing inflammation (NF-κB) and simultaneously activating Scleraxis in an inflammatory environment of tenocytes.

### 2.3. Calebin A Suppresses T-Lymphocyte-, TNF-β-, or TNF-α-Promoted ECM Degradation and Signaling Gene Expression, Similar to IKK Inhibitor (BMS-345541) in Tenocytes

To further investigate previous results, suggesting CA to suppress NF-κB translocation into the nucleus and down-regulation of tendon-specific transcription factor Scleraxis, we sought to examine whether CA has the capacity to prevent cytokine (TNF-α, TNF-β) or T-lymphocyte induced ECM inhibition by analyzing ECM-specific gene expression (Collagen I, Tenomodulin) in tenocytes.

Samples used for Western blot analysis, consisted of tenocytes cultured by themselves with and without CA treatment (basal controls), or tenocytes grown in co-culture with T-lymphocytes either left untreated or treated with CA (1, 5, 10 µM), treated with cytokines (TNF-α, TNF-β) alone, or concomitantly treated with cytokines and CA (5 µM). Additionally, a sample of tenocytes cultured together with T-lymphocytes and treated with BMS-345541 was analyzed. BMS-345541 is a selective inhibitor of IKK subunits, and thus is able to specifically block NF-κB [55]. Therefore, it was subjected as a control of NF-κB inhibition in the experiment.

**Figure 4:**
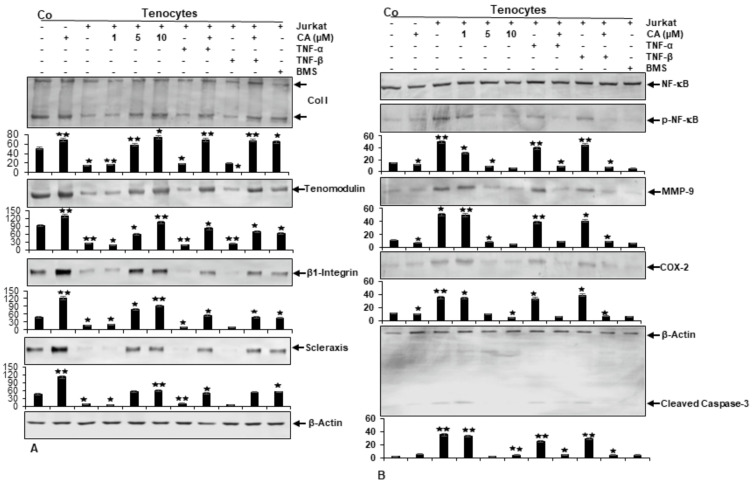
Effect of Calebin A (CA) and IKK-inhibitor BMS-345541 on extracellular matrix and inflammation-associated proteins in tenocyte monolayer cultures. Serum-starved tenocytes in monolayer cultures alone (Co) or co-cultured with T-lymphocytes, or co-cultured with T-lymphocytes and TNF-α or TNF-β (10 ng/mL), were either left untreated or treated with various concentrations of CA (1, 5, 10 µM) or BMS-345541 (5 µM) for 18 h. Western blot analysis was conducted using whole-cell extracts separated by SDS-page, analyzed for (A) Collagen I, Tenomodulin, β1-Integrin, Scleraxis and (B) NF-κB, p-NF-κB, MMP-9, COX-2 and Cleaved Caspase-3, whereby β-Actin served as an internal loading control. Bars demonstrate the mean values of antibodies with the standard deviations of three independent experiments. Values *p* < 0.05 (*) and *p* < 0.01 (**) are considered statistically significant in reference to control.

Immunoblotting results revealed clearly enhanced expression levels of Collagen I, Tenomodulin, β1-Integrin and Scleraxis in cultures treated with CA, similar to BMS-345541-treated culture (Figure 4A). In contrast, untreated tenocyte cultures in an inflammation-induced environment showed a strong down-regulation of tendon-specific protein expression. Together, these results do not only indicate the strong ability of CA to suppress T-lymphocyte- or cytokine-promoted ECM inhibition in an inflammatory environment, but reveal the inhibition of NF-κB as a major target mechanism of CA to unfold its anti-inflammatory and matrix-stimulating effects in tendinitis-like conditions.

### 2.4. Calebin A Suppresses T-Lymphocyte-, TNF-β-, or TNF-α-Promoted NF-κB Activation and NF-κB-Regulated Pro-Inflammatory and Matrix-Degrading Gene Products, Similar to IKK Inhibitor (BMS-345541), in Monolayer Tenocyte Cultures

To gain a deeper insight into previous results suggesting CA could inhibit the activation of NF-κB and its translocation into the nucleus, which is known to stimulate inflammatory processes and up-regulation of catabolic events [32,34,35], we sought to examine the capacity of CA to block inflammation-triggered NF-κB activation in particular, as well as NF-κB promoted gene products taking part in matrix degradation (COX-2, MMP-9) and apoptosis (Cleaved Caspase-3). To investigate if CA suppresses NF-κB activation, tenocyte cultures were analyzed for the phosphorylated form of p65-NF-κB subunits. Immunoblotting revealed higher levels of activated NF-κB expression in the cytokine- and T-lymphocyte-induced inflammatory environment compared to normal control (Figure 4B). Interestingly, in cultures treated with CA, the expression of phosphorylated NF-κB was significantly down-regulated in a concentration-dependent way. In agreement with this observation, NF-κB promoted gene products (MMP-9, COX-2, Cleaved Caspase-3) and showed a decreasing expression level with an increasing CA treatment concentration in inflammation-induced tenocyte environment (Figure 4B). These findings underline previous results and suggest that CA acts as an inhibitor of NF-κB activation by exerting its anti-inflammatory effects, further suppressing the NF-κB pathway-dependent, inflammation-associated (COX-2), matrix-degrading (MMP-9) and apoptosis-related (Cleaved Caspase-3) gene products in an inflammatory tenocyte environment.

### 2.5. Calebin A Suppresses T-Lymphocyte- or TNF-β-Promoted Phosphorylation and Degradation of IκB-α and Phosphorylation of NF-κB in a Time-Dependent Manner in Monolayer Tenocyte Cultures

**Figure 5:**
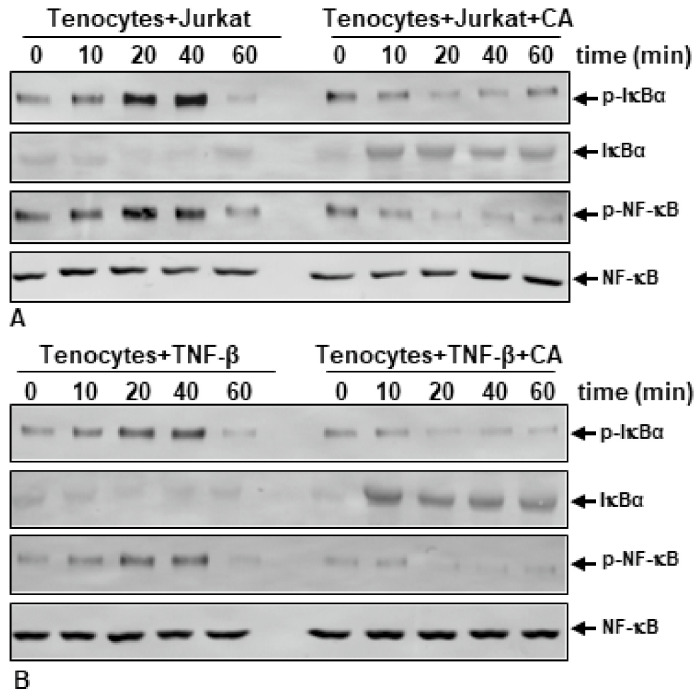
Time-dependent effect of Calebin A (CA) on NF-κB, p-NF-κB, IκBα and p-IκBα in tenocyte monolayer cultures under inflammatory conditions. Serum starved tenocytes (**A**) co-cultured with T-lymphocytes or (**B**) cultured with TNF-β (10 ng/mL) were either left untreated or treated with CA (5 µM) for 0, 10, 20, 40 or 60 min. Whole cell extracts were separated by SDS-PAGE and analyzed via Western blot.

As mentioned before, the transcription factor NF-κB plays a fundamental role in inflammatory processes by further activating pro-inflammatory, matrix-degrading and apoptosis-promoting genes in tenocytes. As a key regulator of NF-κB signaling pathway activation, the IκB-kinase (IKK) complex phosphorylates NF-κB inhibitor IκBα. The phosphorylation process of IκBα is caused by the transcription factor NF-κB, which subsequently is able to translocate in its active form (p-NF-κB) into the nucleus, where it activates various gene expressions [56,57]. To further elucidate the effect of CA on IκBα-mediated NF-κB activation in tenocytes, residing in an inflammatory environment, we performed a Western blot analysis of monolayer cultures, which were either treated with TNF-β or with TNF-β and CA, or were co-cultured with T-lymphocytes and left untreated or treated with CA. Cultures were exposed to their treatments for indicated times and IκBα and NF-κB expression was analyzed (Figure 5). T-lymphocyte- (Figure 5A) and TNF-β (Figure 5B)-induced inflammatory environment cultures similarly showed an increasing expression of the phosphorylated form of IκBα (p-IκBα) and activated p-NF-κB with a maximum level at 40 min, while the expression of NF-κB inhibitor IκBα synchronously decreased with a minimum at 40 min, visualizing the IκBα-mediated NF-κB activation cascade. In inflammation-induced cultures, the expression of p-IκBα and p-NF-κB is significantly suppressed by CA treatment in a time-dependent manner, indicating that CA inhibits the degradation of IκBα and simultaneously blocks pro-inflammatory p-NF-κB activation.

### 2.6. Calebin A Prevents Tendinitis Microenvironment-Triggered Degradation of Extracellular Matrix, β1-Integrin, and Scleraxis Similarly to a Targeted IKK Inhibitor (BMS-345541) in Alginate-Cultured Tenocytes

**Figure 6:**
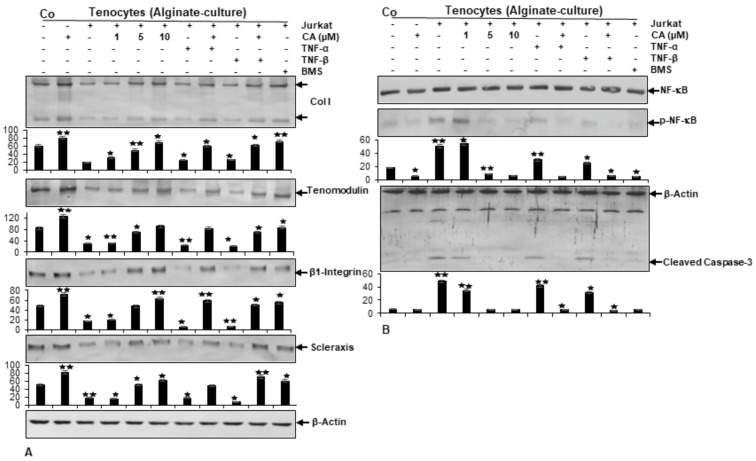
Effect of Calebin A (CA) and specific IKK-inhibitor BMS-345541 on (**A**) extracellular matrix by using antibodies against Collagen I, Tenomodulin, β1-Integrin, Scleraxis and on (**B**) inflammation and apoptosis by using antibodies against NF-κB, p-NF-κB and Cleaved-Caspase-3. Serum starved tenocytes in alginate bead cultures alone (Co) or cultured in TN-ME or co-cultured with T-lymphocytes and TNF-α or TNF-β were either left untreated or treated with various concentrations of CA (1, 5, 10 µM) or BMS-345541 (5 µM) for 8 days. As an internal loading control anti-β-Actin antibody was used. Additionally, densiometric analysis of protein expression found by Western blotting was conducted in triplicate, whereby values *p* < 0.05 (*) and *p* < 0.01 (**) mark statistically significant data compared to control.

To further investigate previous observations, suggesting CA to suppress translocation of NF-κB into the nucleus and T-lymphocyte-, TNF-β-, or TNF-α-promoted ECM degradation, under in vivo like conditions, we created a 3D-multicellular tendinitis microenvironment consisting of tenocyte alginate beads cultured by themselves (basal control) or in pro-inflammatory TN-ME cultures (fibroblasts and T-lymphocytes). For comparison of TN-ME and the role of pro-inflammatory cytokines in TN-ME, some experiments were performed with TNF-a or TNF-β (10 ng/mL) instead of T-lymphocytes. Tenocyte alginate cultures were either left untreated or treated with various concentrations of CA (1, 5, 10 µM) or TNF-α or TNF–β (10 ng/mL) or with BMS-345541 or with CA (5 µM) concomitant with cytokine (TNF-α, TNF-β) treatment (10 ng/mL). The protein expression pattern revealed that CA treatment on TN-ME cultures markedly elevated levels of tendon-specific transcription factor Scleraxis and ECM signaling genes (Collagen I, Tenomodulin, β1-Integrin) in a concentration-dependent manner, similar to CA-treated basal control (Figure 6A). This observation suggests that CA positively affects the tendon- as well as the ECM-specific marker genes expression.

We next wanted to determine whether there was a dependence between the ECM, Scleraxis, and NF-κB signaling pathways. For this purpose, we used BMS-345541, a highly selective inhibitor of the IKK protein pathway for NF-κB signaling. Interestingly, BMS-345541 treatment of tenocytes in TN-ME induced the pattern of expression of Collagen I, Tenomodulin, β1-Integrin, and Scleraxis (Figure 6A), similar to CA. Overall, these findings suggest that the inflammation-protective effect of CA is partially associated with upstream inhibition of NF-κB signaling and concomitant stimulation of Scleraxis.

### 2.7. Calebin A Suppresses Tendinitis Microenvironment-Promoted Elevation of NF-κB, NF-κB-Regulated Matrix-Degrading and Apoptotic Proteins Similarly to a Targeted IKK Inhibitor (BMS-345541) in Alginate-Cultured Tenocytes

To test whether CA would inhibit the TN-ME-induced activity of the pro-inflammatory transcriptional master NF-κB, TN-ME cultures were assayed for the phosphorylated form of p65-NF-κB. The findings showed that activation of p65-NF-κB was significantly increased in TN-ME cultures in comparison to the basal control. CA significantly suppressed TN-ME-stimulated phosphorylation of p65 subunits in tenocytes in a dose-affected manner (Figure 6B). For further investigation of the repressive effects of CA in the TN-ME-promoted NF-κB signaling pathway, we have examined IκBα stimulation triggered by TN-ME as a prerequisite for p65-NF-κB activation. Because phosphorylation and depletion of IκBα result from IKK activation, the role of BMS-345541, a directional IKK suppressor, on TN-ME-promoted IKK activity was also examined. Immunoblotting results showed that TN-ME induced p65-NF-κB phosphorylation in tenocytes. The immunoblotting results in Figure 6B show that CA is able to suppress TN-ME-induced p65-NF-κB activation in tenocytes in the same intensity as BMS-345541 does. Validation of the blots underlines the tremendous anti-inflammatory effect of CA observed in TN-ME, which is selectively affecting NF-κB. Moreover, the expression of NF-κB-mediated proteins included in inflammatory and destructive processes (Cleaved Caspase-3) was remarkably increased in TN-ME cultures in comparison to the basal control (Figure 6B). Treatment with CA resulted in a dose-dependent decrease in the levels of Cleaved Caspase-3 in these cultures (Figure 6B), similar to that observed with BMS-345541 treatment. Densitometric evaluation of immunoblotting results approved the dose-dependent down-regulation of NF-κB, and Cleaved Caspase-3 in tenocytes in TN-ME cultures treated with CA or BMS-345541 (Figure 6B). Overall, these associations seem to indicate that TN-ME promotes tenocyte degradation in part via the NF-κB cascade, which can be specifically blocked by CA, similar to IKK-inhibitor BMS-345541.

### 2.8. Calebin A Suppresses T-Lymphocyte-Promoted p-NF-κB-p65 Association with Scleraxis, Comparable to IKK Inhibitor (BMS-345541) in a Multicellular Tendinitis Microenvironment

**Figure 7:**
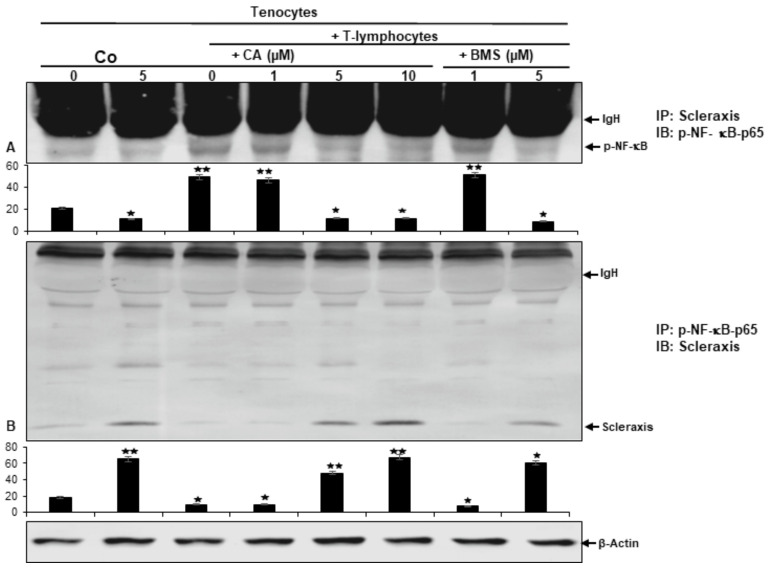
Effects of Calebin A (CA) and BMS-345541 on the association of p-NF-κB to Scleraxis in tenocytes cultured in TN-ME by co-immunoprecipitation assay analysis. Tenocytes in alginate beads cultured solely (Co), were left untreated or treated with CA (5 µM) or co-cultured with fibroblasts and T-lymphocytes (TN-ME) and either kept untreated or treated with different concentrations of CA (1, 5, 10 µM) or BMS-345541 (1, 5 µM) for 8 days. Proteins of samples were either immunoprecipitated (IP) with Scleraxis and immunoblotted (IB) with anti-p-NF-κB (**A**) or immunoprecipitated with anti-p-NF-κB (IP) and subjected to immunoblotting (IB) by using Scleraxis antibody (**B**). As a housekeeping gene, β-Actin was used as a control. Data revealing *p* < 0.05 (*) or *p* < 0.01 (**) are considered as statistically significant.

In the present work TN-ME triggered activation of NF-κB and its signaling pathway end products in tenocytes has been shown to be associated with the down-regulation of Scleraxis. Inversely, CA treatment leads to down-regulation of p65-NF-κB, thereby to up-regulation of Scleraxis, indicating that both transcription factors may act in tandem. To examine the potential functional correlation between Scleraxis and NF-κB in the inflammatory TN-ME, a co-immunoprecipitation assay was performed (Figure 7). Here, tenocyte alginate beads cultured by themselves with or without CA treatment were used as basal controls. In addition, TN-ME cultures were either left untreated or treated with CA or IKK inhibitor BMS-345541, as described in Material and Methods. The samples had been immunoprecipitated with p65-NF-κB-antibody and were subsequently immunoblotted using Scleraxis-directed antibodies and vice versa. As shown in Figure 7, we found that tenocytes treated with CA, similar to BMS-345541, clearly showed a concentration-dependent co-immunoprecipitation of p65-NF-κB with Scleraxis. Contrarily, in untreated basal control cultures only weak co-immunoprecipitation of both transcription factors has been found. Taken together, these data suggest that CA suppresses the T-lymphocyte-promoted increase in p-NF-κB-p65 proteins and decrease in Scleraxis, indicating their association to act in favor of Scleraxis comparable to the effects of IKK inhibitor (BMS-345541) in TN-ME cultures, stimulating tendon proliferation and viability. This finding implicates for the first time, that the natural compound CA specifically targets and modulates the pro-inflammatory NF-κB pathway by its inhibition, in tandem leading to remarkable up-regulation of Scleraxis in a multicellular inflammatory tenocyte environment.

## 3. Discussion

With the aim to explore the effect of Calebin A on T-lymphocyte or TNF-α/TNF-β-triggered inflammation and NF-κB pathways activation as well as on tendon-specific transcription factor Scleraxis as potential targets, together with its underlying mode of action in vitro we created a multicellular tendinitis environment, consisting of fibroblasts, T-lymphocytes and tenocytes.

**Figure 8:**
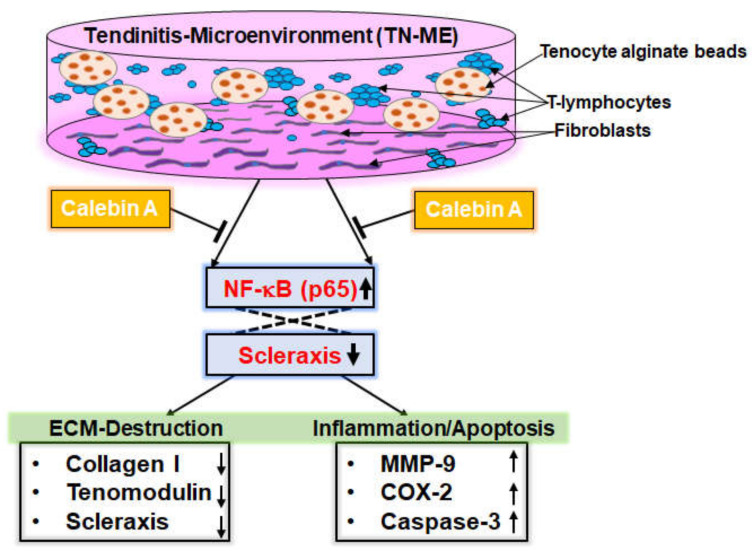
Schematic representation of Calebin A’s mediated anti-inflammatory activity by modulation of the NF-κB/Scleraxis axis in the TN-ME, leading to suppression of pro-inflammatory pathways.

The main novel findings within the present study were: (I) TNF-β-, similar to TNF-α-induced adhesiveness of T-lymphocytes to tenocytes in an inflammatory environment was significantly reduced by CA; (II) T-lymphocyte-triggered NF-κB activation/nuclear translocation as well as down-regulation of Scleraxis was strongly suppressed by CA; (III) T-lymphocyte-, similar to TNF-β- or TNF-α-induced ECM degradation was suppressed by CA, likewise to NF-κB inhibition by using an IKK inhibitor (BMS-345541) in tenocytes; (IV) Furthermore, NF-κB-regulated pro-inflammatory and matrix-degrading gene products, promoted by T-lymphocytes, similar to TNF-β or TNF-α, are down-regulated by CA; (V) CA inhibits phosphorylation and degradation of IκBα and simultaneously blocks p65-NF-κB activity in a time-dependent manner; (VI) Up-regulation of NF-κB and NF-κB-dependent matrix degrading, pro-apoptotic gene products is markedly suppressed by CA, similar to IKK inhibitor (BMS-345541), thereby protecting ECM, β1-Integrin and Scleraxis from degradation in tenocytes; (VII) Finally, CA targets NF-κB/Scleraxis signaling pathway to alleviate inflammation in a TN-ME, similar to IKK inhibitor (BMS-345541). Overall, CA was able to markedly attenuate inflammation and tenocyte ECM degradation in TN-ME by up-regulation of Scleraxis and concomitant inhibition of the NF-κB signaling pathway (Figure 8).

As tendinopathies are painful, disabling and multifactorial conditions, commonly triggered by mechanical overuse, trauma, degenerative processes, drug treatment like quinolone therapy or inflammation [2,25,58], new safe and effective strategies are needed due to limited treatment capacity. Standard treatment therapy, comprising NSAIDs and glucocorticoids, not only show undesirable adverse effects but lack efficacy in long-term treatment because of the limited regeneration capability of tendon due to poor vascularization and hypocellularity [8,10,59,60,61]. Tendinopathies are mostly come along with inflammation-associated destruction of ECM, leading to tendon destruction and decreased biomechanical functionality, whereby pro-inflammatory cytokines as well as catabolic enzymes are mostly up-regulated by NF-κB activity [34,35], which has been previously shown to be suppressed by CA in tumor cells and cells of the connective tissue, but not in tenocytes yet [49,51,52,53].

Our findings showed that CA treatment markedly reduced cytokine-promoted adhesion of T-lymphocytes to tenocytes, which is consistent with previous studies showing that pro-inflammatory cytokines such as TNF-α and TNF-β significantly increase the attachment of T-lymphocytes to other connective tissue cells such as chondrocytes. Since T-lymphocyte-adhesion leads to further stimulation of the inflammatory milieu [62], its interruption appears to be an important target to attenuate inflammation. In addition, we further found that adhesion-targeting of CA is closely associated with down-regulation of T-lymphocyte-promoted pro-inflammatory transcription factor, NF-κB expression by suppressing its phosphorylation and nuclear translocation, and with the concomitant up-regulation of the tendon-specific transcription factor Scleraxis, indicating NF-κB as a major target of CA’s anti-inflammatory actions. With this novel finding, demonstrating that CA strongly inhibits TN-ME-induced decrease in tenocyte vitality and proliferation as well as down-regulation of Scleraxis, while protecting tenocytes ECM from degradation in the same manner as the IKK inhibitor BMS-345541, that specifically blocks NF-κB phosphorylation, does, we clearly detected NF-κB to be strongly implicated in TN-ME-induced inflammatory and matrix-degrading processes. These outcomes are in agreement with previous research, demonstrating that NF-κB signaling is one of the key pro-inflammatory pathways in tendinitis, mediating the activation of several inflammatory and catabolic proteins that are directly linked to the pathogenesis of tendinitis [25,63,64]. Furthermore, we also demonstrated that TN-ME and pro-inflammatory cytokines induce the activation of matrix-degrading (MMP-9), inflammatory (COX-2) and pro-apoptotic (Cleaved Caspase-3) proteins mediated by NF-κB and that their stimulation can be reversed by CA treatment, similar to treatment with an IKK inhibitor (BMS-345541).

These outcomes identify NF-κB as a major target of CA for its anti-inflammatory and matrix-protective effects in tendinitis, corresponding to previous observations demonstrating that the TNF-β- or TNF-α-promoted activity of NF-κB- and NF-κB-dependent end products is down-regulated by polyphenols in cells of various connective tissues, showing inflammation-protective properties by specifically targeting NF-κB [44,65,66,67,68,69]. Against this background, it can be assumed that the NF-κB signaling pathway could be a key treatment target for alleviating inflammation and inflammation-associated processes to support tendon healing.

Moreover, our results showed that CA does not only down-regulate NF-κB-dependent MMP-9, COX-2 and Cleaved Caspase-3, which are known to be involved in tissue destructive processes [25,70], but concurrently stimulates the expression of Collagen type I and β1-Integrin, which are essential components and cell receptors of the tenocytes ECM. β1-Integrin receptors are crucial for the vitality and survival of tenocytes and immensely important for the tendon’s integrity [24,26,28].

In addition, the tendon-specific transcription factor Scleraxis, which is essential for tenocyte differentiation, proliferation and matrix synthesis, as well as Tenomodulin, promoting proper tendon maturation and collagen fibrils adaptation, are key components in tendon healing [71,72]. Both were significantly up-regulated by CA treatment in a concentration-dependent manner, comparable to BMS-345541. This further highlights the protective effect of CA on the tenogenic capacity to proliferate and regenerate by suppressing the TN-ME-induced down-regulation of ECM and key tendon-specific proteins through the inhibition of NF-κB.

The important role of NF-κB as a master regulator of inflammation, being involved in modulation of tendinopathies and tendon healing, was previously reported in several studies [63,73,74]. Under physiological conditions, NF-κB is bound to its inhibitory protein (IκBα) in the cytoplasm and is held in latency therein. Inflammatory stimulation triggers such as pro-inflammatory cytokines lead to the recruitment of the IκB-kinase complex that phosphorylates IκBα. The phosphorylation-activated degradation of IκBα induces NF-κB activation, which is then set free to translocate in its active form (p65-NF-κB) into the nucleus, where it finally up-regulates the transcription of inflammation-associated proteins that contribute to matrix degradation and apoptosis in tenocytes [34,56,63,68,75]. In light of this, the importance of targeting NF-κB to modulate its activation is emphasized, and interestingly, in our study, we demonstrated that CA is able to suppress T-lymphocyte-induced signaling, similar to TNF-β-induced signaling, and the IκBα-mediated activation of NF-κB in a time-dependent manner in TN-ME. This finding is in line with previous research showing similar effects of CA [49,52], underlining again its great multi-modulatory potential to suppress inflammation in catabolic events in tendinitis by specifically inhibiting IκBα-mediated NF-κB activation.

Finally, for the first time, we found evidence that the tendon-specific transcription factor Scleraxis, a marker of tendon viability and tenogenic activity, is stimulated by CA, while NF-κB expression is concomitantly down-regulated in an inflammatory environment, corresponding to the effects of the IKK inhibitor, BMS-345541. As shown in the co-immunoprecipitation assay, this finding demonstrates that both transcription factors functionally act in tandem through the treatment of the CA, indicating a multi-modulatory effect of the natural compound to link inflammation- and tenogenic-related processes in a multicellular tendinitis environment. This further shows the potential of CA to modulate tenogenic maintenance and inflammation in tendinitis by using the NF-κB/Scleraxis axis (Figure 8).

## 4. Materials and Methods

### 4.1. Antibodies, Cytokines and Reagents

TNF-α and antibodies against p65-NF-κB, phospho-p65-NF-κB, Matrix metalloproteinase 9 (MMP-9) and Cleaved-Caspase-3 were purchased from R&D Systems (Heidelberg, Germany). Anti-β-Actin, anti-β1-Integrin, BMS-345541, DAPI (4′,6-diamidino-2-phenylindole), MTT reagent (3-(4,5-dimethylthiazol-2-yl)-2,5-diphenyltetrazolium bromide) and alginate were obtained from Sigma Aldrich (Taufkirchen, Germany); TNF-β was from eBioscience (Frankfurt, Germany); antibodies against IκBα and phospho-IκBα were from Cell Technology (Beverly, MA, USA); and anti-Cyclooxygenase-2 (COX-2) antibody was purchased from Cayman Chemical (Ann Arbor, MI, USA). Polyclonal anti-Tenomodulin was obtained from Santa Cruz Biotechnology (Santa Cruz, CA). Polyclonal anti-Scleraxis was obtained from Abcam PLC (Cambridge, UK). Polyclonal anti-Collagen type I, and alkaline-phosphatase-linked sheep anti-mouse and sheep anti-rabbit secondary antibodies for immunoblotting were purchased from Millipore (Schwalbach, Germany). Secondary rhodamine-coupled antibodies used for fluorescence labeling were purchased from Dianova (Hamburg, Germany).

Calebin A was a noble gift from Sabinsa Corporation (East Windsor, NJ, USA), which was diluted as 5000 µM stock in DMSO (dimethyl-sulfoxide). For experiments, final concentrations were diluted in cell culture medium, whereby the concentration of DMSO never exceeded 0.1%.

Growth medium (Dulbecco’s medium/Ham’s F-12 (1:1) containing 10% fetal bovine serum (FBS), 1% glutamine, 1% penicillin/streptomycin solution (10.000 IU/10.000 IU), 75 μg/mL ascorbic acid, 1% essential amino acids, and 0,5% amphotericin B solution) was acquired from Seromed (Munich, Germany).

### 4.2. Cell Lines and Cell Culture Conditions

Tenocytes were isolated from canine tendon tissue biopsies from tendon-rupture surgery with fully informed owner consent and ethical project approval from the ethical committee of the Ludwig-Maximilians-Universität (Munich, Germany). Cells were grown in a cell culture flask in 10% FBS growth medium until 70% confluency and passaged up to two times, so that passages 2 or 3 were used for experiments. In addition, tenocytes were washed three times with and incubated in serum-starved medium (3% FBS) for 30 min, before being used in the experiments. The MRC-5 fibroblast cell line, which was purchased from the European Collection of Cell Cultures (Salisbury, UK), was grown as monolayer in 12-well plates in growth medium for 24 h, before being used in the assembly of the TN-ME.

Jurkat cells, a T-lymphocyte cell line that was obtained from the Leibniz Institute (Braunschweig, Germany), were cultured in a suspension in 10% FBS growth medium. For all experiments, serum-starved medium (3% FBS) was used.

### 4.3. Experimental Set-Up

A: Tendinitis microenvironment culture

A multicellular tendinitis micro-environment (TN-ME) was established by co-culturing fibroblasts (MRC-5), T-lymphocytes (Jurkat cells) and tenocytes embedded in alginate beads to investigate the effects of CA on the inflammatory condition found in tendinitis, in an set-up similar to in vivo. To create the TN-ME, tenocyte alginate beads were transferred to 12-well plates containing fibroblasts monolayers (3.000/cm^2^) with Jurkat cells in suspension (10.000/mL) in serum-starved medium (3% FBS). Co-culturing was conducted for 8 days. To compare the multicellular TN-ME with the impact of pro-inflammatory cytokines in TN-ME, further experiments were performed with TNF-α or TNF-β (10 ng/mL) instead of T-lymphocytes. In addition, to evaluate the impact of Calebin A on IκBα-mediated NF-κB activation in a time-dependent manner, another set of experiments comprising tenocyte alginate beads were cultured either with inflammation-inducing TNF-β (10 ng/mL) or T-lymphocytes, which were kept untreated or treated with CA (5 µM) for 0, 10, 20, 40, 60 min.

B: Alginate culture

An alginate–tenocytes suspension (1 × 10^6^ cells/mL alginate) was dropped into sterile CaCl2 solution (100 mM) for polymerization, as described in detail in [76,77]. Afterwards, polymerized alginate beads were washed (NaCl, 10% FBS growth medium) before incubation in a serum-starved medium for 1 h. For conducting experiments, the TN-ME cultures were either treated with TNF-α or -β (10 ng/mL), or with BMS-3455541 (5 µM) or with different concentrations of CA (1, 5, 10 µM) alone or with TNF-α or -β (10 ng/mL) combined with CA (5 µM) treatment. Control tenocyte alginate beads were cultured without a TN-ME (i.e., fibroblasts and T-lymphocytes) and left untreated or treated with CA (5µM).

### 4.4. Adhesion Assay of Tenocytes with T-Lymphocytes

To examine interactions between tenocytes and T-lymphocytes, an adhesion assay was conducted as outlined in detail before [65]. Briefly, tenocytes were grown as monolayers (20.000/mL), either by themselves (control) or in T-lymphocyte co-cultures (10.000/mL), and left untreated or treated with various concentrations of CA (1, 5, 10 µM) or with TNF-α or -β (10 ng/mL), or BMS-345541 (5 µM) or with TNF-α and -β (10 ng/mL) concomitant with CA (5 µM). After treatment, cells were incubated for at least 4 h. Subsequently, differently treated co-cultures were photographed at different time slots (12, 18, 24 h) using a light microscope (Zeiss, Jena, Germany). A statistical analysis of T-lymphocytes adhered on tenocytes was conducted by counting 5 microscopic fields.

### 4.5. Western Blot Analysis

Tenocyte samples for Western blotting were derived from the adhesion assay as well as from alginate cultures, both previously described in detail [65,76]. In case of the adhesion assay, tenocyte monolayer was washed with Hank’s solution buffer and removed in hypotonic lysis buffer by gently scraping cells off. Whole-cell lysates were incubated on ice for 15 min and centrifuged for 30 min before their proteins could finally be extracted.

To obtain the protein extract from tenocytes embedded in alginate beads, alginate samples were dissolved as described before [76].

Proteins of samples were separated via SDS-PAGE and transferred onto nitrocellulose membranes (Biorad transblot apparatus). Firstly, membranes were incubated in blocking buffer (5% skimmed milk powder in PBS, 0.1% Tween 20), then with primary antibodies overnight, and finally with secondary antibodies for 120 min the next day. Specific antigen-antibody complexes were identified with nitroblue tetrazolium and 5-bromo-4-chloro-3-indoyl-phosphate (VWR; Munich, Germany). Density measurement was carried out using “Quantity One” software (Bio-Rad, Munich, Germany).

### 4.6. Immunofluorescence Analysis

Tenocytes were seeded on small glass slides (10.000/slide) and grown as monolayers in 10% FBS medium for 24 h. Tenocytes were either grown solely or in co-cultures with T-lymphocytes (0.01 Mio./mL) and kept untreated or treated with CA (5 µM). After 5 h of treatment, slides were fixated in methanol for 30 min. Immunofluorescence investigation of protein expression (NF-κB, Scleraxis) was conducted as previously described [25]. Briefly, cells were incubated with primary antibodies overnight, with secondary rhodamin-linked antibodies for 90 min, and with DAPI for another 15 min the following day, before embedding the slides in Fluoromount (Sigma-Aldrich Munich, Germany). Images of microscopic slides were taken using the Leica DM2000 microscope (Wetzlar, Germany) and statistical analysis was conducted by counting cells in 10 microscopic fields.

### 4.7. Immunoprecipitation

Immunoprecipitation was performed on tenocyte alginate cultures as described in detail [78]. TN-ME cultures were either left untreated or treated with CA (1, 5, 10 µM) or BMS-345541 (1, 5 µM). Basal controls solely grown tenocyte alginate beads, which were left without treatment or treated with CA (5 µM). Pre-cleared cell extracts were blotted, as described in the “Western blot analysis” section, after separation via SDS-PAGE.

### 4.8. Statistical Analysis

Our experiments were performed three times as individual assays. A Wilcoxon–Mann–Whitney test was used for statistical analysis. The results were represented as mean + SD or SEM and compared by one-, two- or three-way ANOVA using SPSS Statistics if the normality test was passed (Kolmogorov–Smirnov test); a value of *p* < 0.05 shows statistically significant deviations.

## 5. Conclusions

Previous research that outlined the necessity of Scleraxis for proper tendon survival, viability, development and healing [72,79,80] corresponds to our results that demonstrate, for the first time, that CA may be of great relevance in novel therapeutic strategies in tendinitis treatment as well as its prevention by targeting, at least in part, the tenogenic-stimulating transcription factor Scleraxis via the NF-κB signaling pathway.

## Data Availability

Data presented in this study are available on request from the corresponding author.
